# Characterizing low effort responding among young African adults recruited via Facebook advertising

**DOI:** 10.1371/journal.pone.0250303

**Published:** 2021-05-14

**Authors:** Emmanuel Olawale Olamijuwon

**Affiliations:** 1 Faculty of Social Science, Department of Statistics and Demography, University of Eswatini, Kwaluseni, Eswatini; 2 Demography and Population Studies Programme, Schools of Public Health and Social Sciences, University of the Witwatersrand, Johannesburg, South Africa; University of Florida, UNITED STATES

## Abstract

Multiple studies have successfully used Facebook’s advertising platform to recruit study participants. However, very limited methodological discussion exists regarding the magnitude of low effort responses from participants recruited via Facebook and African samples. This study describes a quasi-random study that identified and enrolled young adults in Kenya, Nigeria, and South Africa between 22 May and 6 June 2020, based on an advertisement budget of 9,000.00 ZAR (US $521.44). The advertisements attracted over 900,000 views, 11,711‬ unique clicks, 1190 survey responses, and a total of 978 completed responses from young adults in the three countries during the period. Competition rates on key demographic characteristics ranged from 82% among those who attempted the survey to about 94% among eligible participants. The average cost of the advertisements was 7.56 ZAR (US $0.43) per survey participant, 8.68 ZAR (US $0.50) per eligible response, and 9.20 ZAR (US $0.53) per complete response. The passage rate on the attention checks varied from about 50% on the first question to as high as 76% on the third attention check question. About 59% of the sample passed all the attention checks, while 30% passed none of the attention checks. Results from a truncated Poisson regression model suggest that passage of attention checks was significantly associated with demographically relevant characteristics such as age and sex. Overall, the findings contribute to the growing body of literature describing the strengths and limitations of online sample frames, especially in developing countries.

## Introduction

### Background

Evidence of the use of Facebook as a sampling frame for studying population processes continues to emerge. Today, a large body of studies have used the digital sampling frame to study Polish migrants [1], young people [2–5], those of low socioeconomic status [6], respondents in hard to reach areas [7–9] and more recently, health behaviors [10] and information-seeking [11] during the outbreak of diseases. These studies, among others, have demonstrated that this method of sampling study participants is moderately successful at collecting representative samples at a relatively low cost [4,12–15] and with non-significant biases [16,17]. Even more, data from online samples can provide insights into the broader issues faced by people online and offline [16].

However, most of these studies are focused mainly on participants in developed countries. There is an increasing need to better understand how Facebook’s advertising platform could be used to recruit participants based in African countries. A bulk of web-based studies in African countries have recruited participants mostly through snowball samples of university students [18–21] and men who have sex with men [22]. In some cases, the recruitment group was comprised of medical practitioners such as physicians [23,24] and public health officials [25].

To my knowledge, this method of sampling participants in Africa from a digital frame has mostly focused on a sample of men who have sex with other men [26], and I am aware of only a few publications using paid Facebook advertising to recruit participants in Africa [27,28]. These studies have all focused on the entire population, and limited evidence abounds about the potentials for recruiting young adults, a group known to have higher access to the internet.

Today, adolescents and young adults have high unmet needs for family planning, mistimed and unwanted pregnancies, sexually transmissible infections, and HIV rates [29–33]. The increasing surge of health problems in this population necessitates further research. Unfortunately, very few national datasets from which to draw valid conclusions exist. Where available, the release is often delayed and may not address the present population’s needs. Unlike in the past, young people increasingly use the internet to seek information and connect with friends and family [34]. This is primarily because social media internet sites like Facebook, Instagram, Twitter, and YouTube are quickly replacing traditional forms of communication since they offer rapid transference of ideas and opinions through a relatively low-cost and user-friendly network [34,35].

Facebook estimates that adolescents and young adults aged 13–24 years account for about 37% of all the users on the network [36]. It is also estimated that there are over four billion internet users globally, coupled with significant increases in mobile phone subscription in developing countries [37,38]. Due to increased mobile internet coverage, many adolescents and young adults in African countries can now connect anywhere with reception regardless of residence type (rural or urban) and level of wealth [34,39]. More so, completing surveys via digital devices may be appealing to this population group, thus leading to higher response rates [40]. Today, very few methodological analyses of web-based recruitment of young adults via Facebook advertisements exist in African countries. As many young African adults connect online and interact with others, there has been a window of opportunity to complement existing survey approaches with samples drawn online to better understand some of the health challenges faced by this population.

In this study, I evaluated the effectiveness and efficiency of recruiting young African adults using the advertising platform. More precisely, I used a series of measures to evaluate the quality of survey responses by checking for multiple attempts from participants and the level of attentiveness to the survey. In the absence of any literature on the potential to reach young African adults via Fakebook’s advertisements, I also validated the performance (advertisement reach, time, and cost) of the advertisement campaigns using direct measures available via the advertising platform.

### Issues in web-based participant recruitment

The digital age offers a promising opportunity for researchers to conduct cutting edge studies, including the ability to effectively recruit a large pool of participants and hard-to-reach populations, as they are relatively cheaper and faster compared to in-person recruitment [12,40]. In addition, surveys delivered via the internet overcome many limitations of in-person recruitment since online respondents may be more likely to provide honest answers and minimize social desirability [41]. This may, in part, be because web-based surveys allow for anonymity since participants are free to withhold their names and they are not personally known to the researchers. Online surveys also do not have errors introduced by interviewers [42].

However, these many benefits come at huge costs, including sampling bias and data quality issues. The lack of a central registration of users on the web is believed to be an important limitation of the web-based survey since achieving a random sample may be unrealistic [40]. However, by imposing demographic quotas that allow the targeting of potential participants on Facebook based on a set of predefined demographic characteristics, researchers can now overcome potential selection biases associated with online surveys [14,43]. In fact, a recent study has shown that self-selection biases on the Facebook platform are negligible [17]. Some scholarships have also recommended using poststratification weights to adjust the sample counts from Facebook based on the extent of its deviation from the general population on important characteristics [44]. Today, much of what remains ensures that responses are meaningfully valid, that participants respond in ways that reflect their true behaviors, and avoid spurious results and conclusions that may arise from poor quality responses from participants who put in less effort when attempting the survey. Since population research is intended to influence policy and practice and ultimately contribute to overall population development, the need for quality data cannot be overemphasized. However, providing high-quality responses requires respondents to devote their attention to completing a questionnaire and, thus, thoroughly assessing every single question. This requirement may particularly be challenging to achieve in online surveys where participants are unsupervised and face significantly higher levels of distraction from several sources [45] or might multitask while completing the survey [46]. Moreover, the format and mode of responding to online surveys make it possible to have extreme forms of satisficing—a term used to describe the cognitive shortcut taken in the process of answering survey questions [47].

As online data sources become more prominent in social science research, there is an increasing need to ensure that data obtained from online surveys are of high quality. To address concerns about careless responding in online or other self-administered surveys, researchers have adopted multiple approaches to effectively gauge participants’ attentiveness, including the use of instructional manipulation checks [48], bogus items, instructed response items [49], logical statements, directed queries, reverse scaling, and response time among others [50]. These questions, placed either in the instructions or intermingled with the questions themselves, are one way to determine whether respondents are paying attention. In recent times, evidence suggests that inattentive responses are becoming a common phenomenon in self-administered surveys. Using a large donor dataset, Abbey and Meloy [50] found that the extent of inattentiveness based on instruction manipulation checks could be as low as 5% and as high as 45%. Between a third and a half of respondents in another national sample also failed to correctly answer an attention check question [51]. Oppenheimer et al. [48] have also suggested that careless responding may be higher among non-motivated samples—participants who are only attracted to a study because an incentive is offered, leading them to rush through the survey to be eligible for an incentive [51,52].

### The use of attention checks in web-based surveys

Attention checks—questions with an obvious correct answer—are increasingly used in social sciences and have garnered intense discussion, in part because it is an efficient, low-cost method of enhancing data quality. These types of questions are incorporated into the survey design to make respondents demonstrate that they have read and processed the survey questions. Perhaps the most common form of attention check is the instructed response items (IRIs) which are items embedded in a scale with an obvious correct answer. Respondents who fail an IRI are consistently more prone to response behaviors that are commonly associated with measurement and non-response errors [53].

Previous studies have focused mostly on how failing attention checks relates to other indicators of poor respondent behavior, such as irrational responses, inconsistent answers, or speeding while completing surveys [48,51,54]. Most notably, the response time metric classifies participants as being overly fast or slow based on distributional or expected timing outcomes. Some prior studies suggest that respondents who devote little effort to processing and answering survey questions can be expected to complete a survey very fast [55]. To substantiate this finding, Gummer et al. [53] found that those who failed the IRI were more likely to speed through the survey than more attentive participants. In another study, participants who failed an instructional manipulation check required less cognition effort and took less time to complete the study experiment than those who passed [48]. However, response time to complete a survey may also be affected by the strength and speed of the internet connection, especially in African countries. This conclusion, and some others, led me to believe that this relationship may emerge differently in a sample of young adults in African countries.

Today, there are diverse perspectives regarding the use of attention checks. While the initial intention was to filter poor response behavior, such as skipping crucial questions, speeding, or other undesirable behaviors, the approach has also been adapted to improve the quality of responses. Attention checks may serve as a warning to careless participants in the survey, and giving warnings can effectively reduce careless responses and reduce statistical noise [48,56,57]. However, some researchers fear that the use of attention checks as warnings may also have undesirable effects like increasing socially desirable responses [45], demotivating participants who may subsequently drop out of the study or cause participants to answer subsequent questions differently, incorrectly or inaccurately [58] because they feel watched or untrusted by study investigators [51]. Attention checks may increase deliberation [58], and deliberation can cause inaccurate or inconsistent responses. The use of attention checks may, therefore, bias survey responses and threaten scale validity [59,60]. However, recent evidence suggests that the inclusion of instructed response items does not pose a threat to scale validity nor influence how participants approach subsequent questions [61]. The finding is further substantiated by a recent study showing that the use of attention checks did not affect response behaviors neither positively nor negatively [53].

Given the increasing evidence of the use of attention check questions, the more important question is how researchers can effectively deal with data from respondents who devote less effort to survey questions. Results have been mixed on whether attention checks accomplish what the researcher intends them to, with some studies emphasizing its benefits and others arguing that removing data based on attention checks produces biased results and threatens external validity. Low-quality responses from participants who devote less effort add noise and can substantially decrease statistical power. Some prior works have reported that excluding inattentive respondents from data analysis reduced statistical noise and increased the efficiency of experiments [48]. Greszki et al. [55] found that the exclusion of too fast respondents did not significantly alter the results of their substantive models. This finding is further substantiated by Anduiza, and Galias [62] and Gummer et al. [53], who found that excluding inattentive participants did not significantly contribute to improvement in the fit of their explanatory models. Gummer et al. [53] further noted that they would have drawn the same substantive conclusions from each of the four models with and without inattentive respondents [53].

A large body of scholarship has also advised against eliminating respondents who did not fully follow the instructions and failed attention checks [51,58,62,63]. Some recent studies have raised a concern that eliminating participants who fail attention checks might lead to a demographic bias, threaten external validity, and limit the generalizability of study findings if participants of a specific demographic are more likely to fail attention checks compared to others [48]. Knowing to what extent failing to pass an attention check is conditioned by age, education, or motivation is essential, but it also has relevant implications for dealing with those respondents who fail attention checks. Oppenheimer et al. [48] did not find any effects of gender, age, self-reported motivation, or material motivation on failing attention checks but attributed the lack of differences to a small sample of students. Other studies have found that passing attention checks were associated with sociodemographic characteristics such as education and race [51].

Despite the increasing popularity of the Facebook advertising platform for recruiting study participants, surprisingly, very few studies exist on the use of attention checks and how they may be used to identify respondents who provide answers with poor quality and less effort. The bulk of studies using attention checks have used samples drawn from non-representative samples [61], large scale panel online (or offline) surveys [53,62], or a comparison of both [45,64–66]. Many of these samples are more experienced in completing surveys, and the data sources are usually of high quality. As samples drawn from digital sampling frames gain rapid interest in social science research, it is also essential to understand the extent of careless responding among participants recruited via the advertising platform. In addition to attention checks as measures of data quality, I also examine the frequency of multiple responses and response time to assess the level of effort devoted by survey participants. Understanding the association between sociodemographic characteristics and pass rates of attention checks is emerging to be an exciting avenue to explore, primarily because demographic differences could pose severe limitations if failures are to be excluded from further analysis. Not only may such inquiry advance the management and implementation of surveys using digital platforms in African countries but also advance the use of attention check questions in social science research and other fields of studies.

### Contribution

The present study contributes to the literature primarily in two ways. First, I assessed the feasibility of recruiting young adults in African countries using Facebook’s advertising platform. My sampling approach allowed me to compare recruitment strategies across three different populations (Kenya, Nigeria, South Africa). I focused on data that permits the measurement of the effectiveness and efficiency of data collection using the advertising platform. Effectiveness is the degree to which the recruitment objectives (quality responses) are achieved. Effectiveness was assessed by exploring the quality of the data collected, including missing data, response time, multiple responses, and pass rates of attention check questions. On the other hand, efficiency is a consideration of cost and the ability to complete data collection in the best possible way, with minimal waste of time and effort. The survey tools provided extensive information about survey respondents, while information about advertisement reach and cost were retrieved from the advertisement manager.

Secondly, I combined multiple approaches using response time and repeated IRIs (here referred to as attention check questions) throughout the survey to assess varying levels of attentiveness to survey questions from a sample of young adults recruited via the Facebook advertising platform [50,62]. I also examined if there were any observable sociodemographic differences between attentive and nonattentive participants. The focus of this study on attention checks as a proxy measure of attention and data quality was based on prior evidence suggesting that its use outperforms other traditional metrics with respect to being able to filter respondents who give “irrational” answers [54]. Where relevant, the methodological issues involved were illustrated with examples from my own practice. This inquiry is therefore critical to advance the use of digital sampling frames in recruiting participants in African countries, especially when there is a pressing need to digitalize survey data collection in African countries.

## Materials and methods

### Overview

The health information survey was a cross-sectional survey of young adults living in Kenya, Nigeria, and South Africa. The overarching aim of this survey was to understand the [sexual] health information needs of young adults in African countries. The research received approval from the University of the Witwatersrand non-medical human research ethics committee. No additional national approval was required nor obtained for this study. Potential participants were instructed to read the information page and the associated answers to some frequently asked questions before initiating the survey. This page included information about participants’ eligibility, study objectives, number of questions, incentives, and my contact information should they require any additional information about the survey. After reading the information statement, potential participants provided formal consent by clicking on an “I agree to participate in this survey, proceed” button. Participants were aware that they could quit the survey at any time should they not wish to complete it. All advertisements on Facebook (https://www.facebook.com/policies/ads/) were reviewed to ensure compliance with Facebook’s guidelines for advertisements and were subsequently approved by the company. Facebook does not reveal the identity of members of the target population, and as such, it is possible to conduct a survey with an anonymous sample.

### Study design and participants

A marketing tool that provides an opportunity to place advertisements was leveraged to reach the target population of young adults aged 18–24 years in African countries over two weeks (22 May-8 June 2020). The study included young adults aged 18–24 years who were residents in Kenya, Nigeria, and South Africa. The selection of these countries was based on the availability of the internet and communicative technologies in these countries and also to provide representation for each sub-region in sub-Saharan Africa [39]. Participants had to be English literate to be eligible for the study, and all the countries are also largely English-speaking.

Facebook’s advertising platform provides not only a recruitment tool but also a sampling frame—especially since no sampling frame of young African adults with access to the internet exists [67]. Although Facebook provides several options to reach a specified audience based on objectives such as brand awareness, reach, engagement, conversions, and traffic, I chose the traffic campaign objective based on evidence of effectiveness in prior studies [67]. According to Facebook, this campaign’s objective is to “send more people to a destination such as a website, app, or Messenger conversation.”

I leveraged an existing Facebook page (Health Information Survey for Young Adults) which was previously created for this study to place advertisements on the Facebook advertising platform. Using this name had the advantage of communicating further the goal of the advertisements, reassuring participants of the legitimacy of the survey, and possibly piquing the interest of potential participants. The advertisements included a show headline, a picture, and a link to the survey website (SHYad.NET). The survey website was optimized for mobile devices such as smartphones and tablets, thereby increasing accessibility for those without a computer. The survey website and all text in the advertisements were in English.

**Fig 1:**
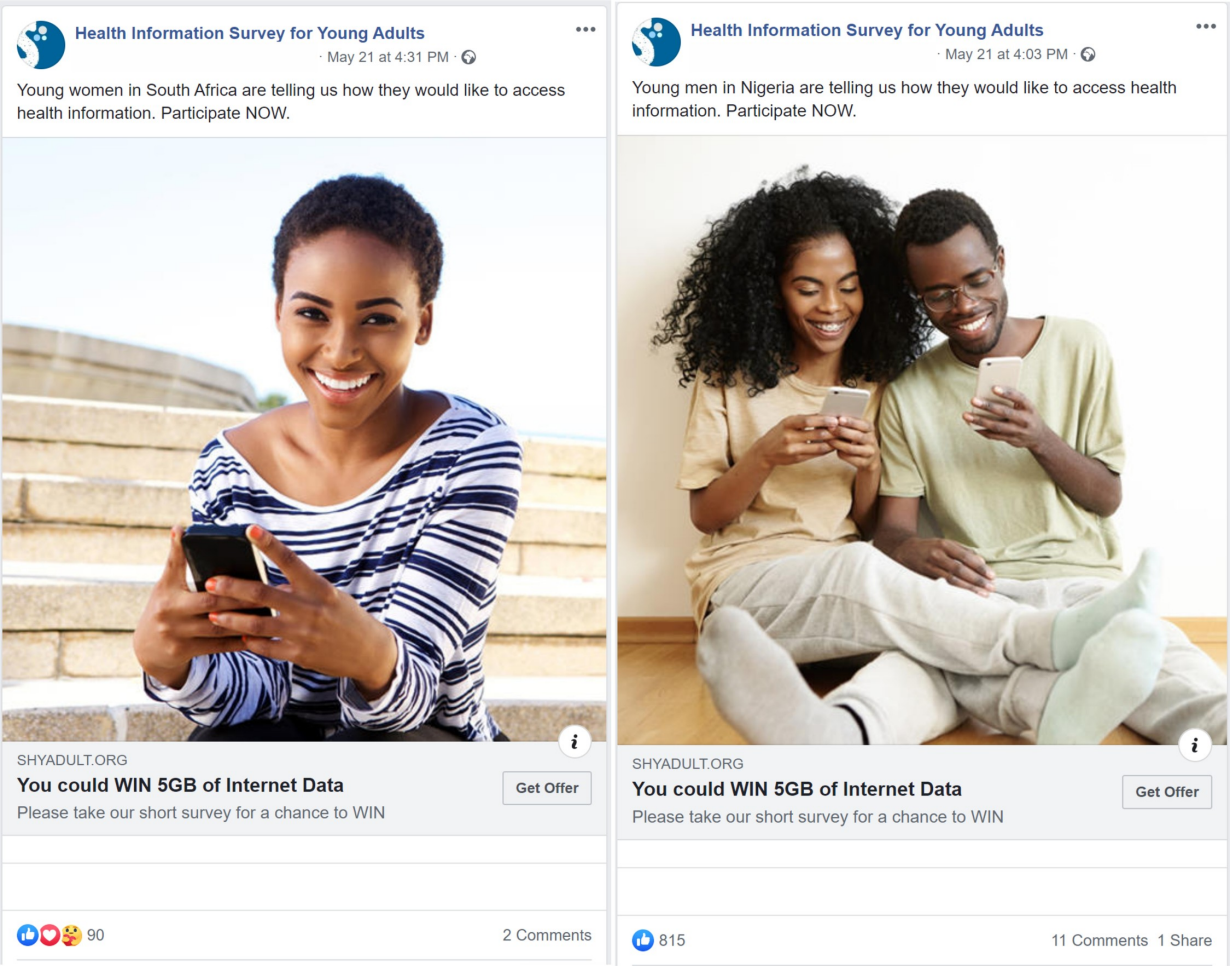
Examples of Facebook advertisements.

The wording of texts and images used in the survey was carefully considered as they were intended to directly motivate potential participants to participate in the survey and reduce the possibility of selection bias such that only those interested in the study were recruited. For each advertisement (see Fig 1), I included the gender and country of the target population (Young [gender] in [country] are telling us how they would like to access health information. Participate NOW). Since pictures used are likely to significantly affect the performance (link clicks) of advertisements [2], I purchased eight stock images of young adults (based on country and gender), including the rights to use them in advertisements. Every advertisement included a headline informing participants that they could win 5GB of internet data if they participated in the survey.

Based on the Facebook algorithm for displaying ads, Facebook is more likely to display ads that receive the most clicks during the learning phases—usually after 50 link clicks. This could result in a homogenous, biased sample if users who share certain sociodemographic or cultural traits are more inclined to click on a Facebook ad than others if they prefer the picture or the texts used [68]. Furthermore, Facebook sampling tends to oversample the better educated, young, and most active potential participants of a demographic cohort [69,70]. As a result, if more highly educated young men in Nigeria engage in click behavior during the learning phase of an advertisement, Facebook is likely to display more advertisements to this group and display fewer ads to a group that is unlikely to click on links. To avoid having a homogenous, biased sample, researchers have advised targeting diverse demographic strata, especially those for which differences are expected, with specific and separate ad sets [14,68]. Because the Facebook population is large and the ad targeting well-developed, it is possible to use quota sampling to generate a sample that corresponds to the general population of one or more demographics—even those who have less than secondary education. Since I expected that participants of different gender, educational levels, and countries of residence would exhibit differences in their willingness to interact with the survey, I generated 12 strata based on different combinations of key sociodemographic characteristics, such as country (Kenya, Nigeria, and South Africa), gender (male and female), and educational attainment (secondary education, and other levels of education). To incentivize participation, participants had a chance to win 5GB of internet data upon completing the survey. Participants who wished to be considered for 5GB internet data were asked to provide a mobile number on which they could be contacted. Each completed survey with an associated mobile number was entered into a draw to win 5GB internet data at the end of the survey. The total cost for the incentive was 1650 ZAR (US $95.60) for six winners—two drawn from each country.

### Campaign settings, reach, and costs

One of the unique features of the Facebook advertising platform is the opportunity for detailed targeting of the desired population. Facebook collects detailed data on the users’ attributes that advertisers can be use to target their campaigns quite precisely. These attributes include the standard demographics such as age, sex, education, location, interests, and behaviors. Facebook targeting options include an opportunity to define audiences, placement, budget, and schedule. In terms of location, advertisers can target users living in or that have recently lived in a specific location, as well as people traveling to a location. Since I was more interested in a national-level analysis of young adults’ responses, the location for each ad set was defined as people living in each of the countries under study. Age was specified as ranging from 18 to 24 years. In the demographic’s category, users were targeted based on whether they were “in high school,” “high school grad,” “attained some high school,” or were not in any of the three categories. A detailed description of the different strata and the potential reach (audience size) is provided in Table 1.

**Table 1:** Facebook’s assessment of the potential reach of advertisements for young adults in Kenya, Nigeria, South Africa.

Location	Male		Female	
	Other Education ^a^	Secondary Education ^b^	Other Education ^a^	Secondary Education ^b^
Kenya	1,600,000	310,000	1,200,000	180,000
Nigeria	4,700,000	710,000	3,500,000	540,000
South Africa	2,000,000	750,000	1,900,000	800,000

*Note*: Variables used to define target populations

Location: People living in this location.

Age: 18–24 Years.

Demographics >> Education >> Educational level.

Languages: English (All).

^a-^Excludes those who indicated that their level of education is “high school”, “high school grad” and “some high school”.

^b-^Includes only those who indicated that their level of education is “high school”, “high school grad” and “some high school.

An automatic placement was chosen for this study, allowing advertisements to be shown to the target population on the feeds, in stories, in-stream, search, messages, in-article, as well as apps and sites. Ads were also shown on Instagram. According to the Facebook advertising platform, Facebook delivery systems allocate the budget for each ad set across multiple placements based on where they are likely to perform best. Futhermore, Facebook provides several options for optimizing the delivery of advertisements. These include optimization for landing page views, link clicks, impressions, and unique daily reach. Each option for optimizing an advertisement has a different objective and although optimizing for landing page view seemed more appropriate for this study, I optimized the advertisements for link clicks which delivers advertisements to those who are most likely to click on the ads. At the time of this analysis, the optimization of advertisements for landing page views involved delivering the advertisements to people who were more likely to click on the advertisements’ links and wait for the website or survey page to be fully loaded. However, since it was unclear how the demographics of those who were likely to click on the link or load the page may differ from those who were not in the target population, this approach might have increased the possibility of selection bias. In addition, optimizing the advertisements for link clicks also implied that payment is only made when a potential participant clicks on an advertisement link, rather than when an advertisement is served or seen by the target population.

The total advertisement budget for the present study was 9,000 ZAR (US $521.44) divided equally across the three campaigns, that is, 3,000 ZAR (US $173.81) per recruitment site. This amount was evenly divided across the three strata (male, female, education (high school, non-high school)). An automatic budget was set for each advertisement, and the cost for the advertisement for each country was automatically determined by Facebook based on biddings by other advertisers. Assuming a proportional to size sampling approach, I allocated the same budget of 900 ZAR (US $52.14) to all strata except the high school educational strata (600 ZAR (US $34.76)). This approach implied that the sample size for each stratum would be dependent on the size of the audience and the cost per link click. Participant recruitment for each stratum ceased once the budget had been exhausted. Performance metrics for each advertisement were obtained from the Facebook Ad Manager.

### Survey instrument

Survey questions were made available via the project website (SHYad.NET). The study being reported here was comprised of two sections—sociodemographic characteristics and quality checks. The sociodemographic section comprised of six questions concentrating on participants’ demographic characteristics (age, sex) and social characteristics (country of residence, educational attainment, race/ethnicity, frequency of internet use). The survey asked participants to indicate their age as at the last birthday and gender (male/female). Three design elements were incorporated in this study to improve the quality of responses:

- The age column in the survey was open-ended, with options as low as 13 and as high as 45 years old. The country column was the same, implying that even those who were not eligible could participate in the survey without providing incorrect responses (age/country) in an attempt to participate in the survey. In addition, the survey included “do not know,” “prefer not to say,” and “not sure” for respondents who did not wish to respond. However, all ineligible participants (including those below 18 years) were carefully removed during data analysis.
- A short description and purpose of the attention check questions were presented on the information page to warn the potential participants and motivate them to provide quality responses.
- I also used attention check questions as prompts to minimize careless responding. All attention checks were short to minimize misleading information [71] and ensure that those who failed were those who had not thoroughly read the instructions and questions.

While many of the approaches in the design element were adapted from previous studies, some were my design. The attention checks comprised of three questions attempting to gauge participants’ attentiveness to the questions. The questions looked like other survey items and were randomly positioned among all survey questions, so that the participants could not guess the position of the last attention check question based on the first two attempts. The checks ostensibly asked respondents to select an option that had the color “grey,” “green,” or “red” (see Appendix in S1 Appendix for exact wording of attention checks). The design and use of multiple attention check questions were primarily because a single question had been deemed ineffective to distinguish inattentive from attentive participants [51] and because it permitted an assessment of variability in passage rates between subjects. Participants who selected the correct category for each question were classified as having passed the attention checks. The number of checks passed was derived from the sum of all attention check questions passed by a participant. The response category for this measure ranged from 0 (passed no attention check questions) to 3 (passed all checks).

Furthermore, the internet protocol address of the device used in participating in the survey was logged to identify multiple responses and possible fraudulent responses that may arise as a result [72]. This was essential since some participants may be tempted to participate in the survey more than once to increase their chances of winning an incentive. In addition, the survey website logged the start and end time for each survey participant.

### Model specification

A count regression model was specified in this study to evaluate the association between the sociodemographic characteristics and the number of attention checks passed. While the Poisson model is well-suited for count data as the attention score in this study, the model does not restrict the upper bound (0 to *inf*) of the distribution, which is unfortunately restricted (at 3) in this study. More precisely, all the participants in this study have a maximum score of 3, and no participants can have a score above 3. As a result, a truncated Poisson regression model censored at three was fitted on the data using the VGAM package in R [73]. Multiple checks for overdispersion showed that the mean and variance of the distribution are not significantly different. The ratio of the residual deviance of the model to its degrees of freedom also provided additional support that the data is not over-dispersed.

Multiple binomial regression models were also specified to delineate associations between the sociodemographic characteristics and the number of attention checks passed at different thresholds. These models serve two primary purposes. First, as a robustness check for the main results, and secondly, to demonstrate how the association between the independent and dependent variables differed according to varying thresholds of the dependent variable. This is particularly because multiple studies have suggested that the threshold for excluding nonattentive participants could lead to demographic bias and pose a significant threat to the validity of the study findings [48].

For this purpose, the main dependent variable was transformed to create three additional dependent variables coded into binary categories across the different thresholds. Participants were categorized as having:

- passed no attention check (0) vs passed at least one attention check (1)
- passed no more than one attention check (0) vs passed at least two attention checks (1).
- passed no more than two attention checks (0) vs passed at least three attention checks (1).

Due to the size of the distributions, an asymmetric link function- complementary log-logit model *(cloglog)* was fitted on each of the transformed outcome variables. Specifically, symmetric link functions are known to inadequately fit binary data when the latent probability of a binary variable approaches 0 at a different rate than it approaches 1, thus leading to bias in the mean response estimates [74,75]. Furthermore, the *cloglog* model is frequently used when the probability of an event is very small or very large. Model fit indices such as the Akaike Information Criterion (AIC) and Bayesian Information Criterion (BIC) index provided evidence that the *cloglog* model was significantly better than other (logit) link functions.

## Results

### Performance of advertising campaign and recruitment results

Table 2 presents conversion rates for the advertisements across the three countries. Over two weeks (22 May-6 June 2020), the advertisement campaign reached over 900,000 young adults in Kenya, Nigeria, and South Africa based on an advertisement cost of about 9,000.00 ZAR (US $521.44). During the period, a total of 11,711‬ unique clicks were observed in the advertisement campaign in all countries ranging from 2,739 link clicks in South Africa to 5,932 link clicks from young adults in Nigeria. Data from the Facebook Pixel directly installed on the survey website revealed about 31% (3,661) conversion of those who landed on the survey website. About 32% of those who landed on the survey website further participated in the survey. About 13% (153) of those who participated in the survey were ineligible, that is, older than 24 or younger than 18 or were not living in Kenya, Nigeria, or South Africa at the time of the survey. This implied a total match rate of about 87% based on advertising target and self-reported demographic characteristics—that is, 87% of those recruited met the inclusion criteria (young and residing in Kenya, Nigeria, or South Africa). A total of 978 completed responses (on demographic characteristics) was received during the recruitment period, implying an 82% completion rate from 1190 participants who attempted the survey and a 94% competition rate from the 1037 eligible participants. To better understand variations in the cost of each ad set, a summary of the ad performance obtained from the Ad Manager is presented in Table 3. The advertising cost per unique link click ranged from 0.25 ZAR among men and women with other levels of education (excluding high school) in Nigeria to about 0.75 ZAR among young women with high school education in Kenya. Overall, the average cost of the advertisements was 7.56 ZAR per survey participant, 8.68 ZAR per eligible response, 9.20 ZAR per complete response, and 14.63 ZAR per attentive (passed 2+ attention checks) participant.

**Table 2:** Summary of advertisement campaigns and results.

Country	Facebook Audience Size ^a^	Advertisement Reach ^a^	Unique Link Clicks ^a^	Landing Page Views ^b^	Survey Participants ^c^	Eligible Participants ^c^	Complete Responses ^c^
Kenya	3,400,000	241,598	3,040	2,000	499	446	421
Nigeria	9,400,000	493,440	5,932	961	391	320	305
South Africa	5,400,000	223,935	2,739	700	300	271	252

a–Metrics obtained from Facebook ads manager.

b–Metrics obtained from Facebook ads manager via pixel.

c–Metrics obtained from survey website (SHYad.NET).

**Table 3:** Summary of Ad. performance.

Country	Ad Text	Target	Impressions	Reach	Unique links click	Cost per unique click (ZAR)	Total amount spent (ZAR)
Kenya	Young women in Kenya are telling us how they would like to access health information. Participate NOW.	Age: 18–24; Gender: Female; Country: Kenya; Education: exc. High school	170,300	102,943	2,069	0.43	899.44 ($52.11)
Kenya	Young women in Kenya are telling us how they would like to access health information. Participate NOW.	Age: 18–24; Gender: Female; Country: Kenya; Education: High school	98,218	42,511	799	0.75	598.53 ($34.68)
Kenya	Young men in Kenya are telling us how they would like to access health information. Participate NOW.	Age: 18–24; Gender: Male; Country: Kenya; Education: exc. High school	226,877	130,015	1,856	0.48	899.63 ($52.12)
Kenya	Young men in Kenya are telling us how they would like to access health information. Participate NOW.	Age: 18–24; Gender: Male; Country: Kenya; Education: High school	103,809	52,864	900	0.67	599.86 ($34.75)
Nigeria	Young women in Nigeria are telling us how they would like to access health information. Participate NOW.	Age: 18–24; Gender: Female; Country: Nigeria; Education: exc. High school	390,352	258,816	3,576	0.25	899.44 ($52.11)
Nigeria	Young women in Nigeria are telling us how they would like to access health information. Participate NOW.	Age: 18–24; Gender: Female; Country: Nigeria; Education: High school	194,359	101,057	1,698	0.35	598.74 ($34.69)
Nigeria	Young men in Nigeria are telling us how they would like to access health information. Participate NOW.	Age: 18–24; Gender: Male; Country: Nigeria; Education: exc. High school	360,991	253,375	3,662	0.25	899.02 ($52.09)
Nigeria	Young men in Nigeria are telling us how they would like to access health information. Participate NOW.	Age: 18–24; Gender: Male; Country: Nigeria; Education: High school	184,285	108,448	1,826	0.33	599.24 ($34.72)
South Africa	Young women in South Africa are telling us how they would like to access health information. Participate NOW.	Age: 18–24; Gender: Female; Country: South Africa; Education: exc. High school	164,245	100,863	1,761	0.51	899.73 ($52.13)
South Africa	Young women in South Africa are telling us how they would like to access health information. Participate NOW.	Age: 18–24; Gender: Female; Country: South Africa; Education: High school	108,603	582,56	853	0.70	599.39 ($34.72)
South Africa	Young men in South Africa are telling us how they would like to access health information. Participate NOW.	Age: 18–24; Gender: Male; Country: South Africa; Education: exc. High school	161,734	929,60	1,449	0.62	898.24 ($52.04)
South Africa	Young men in South Africa are telling us how they would like to access health information. Participate NOW.	Age: 18–24; Gender: Male; Country: South Africa; Education: High school	101,574	56,192	827	0.72	598.78 ($34.69)

### Survey responses quality check

**Fig 2:**
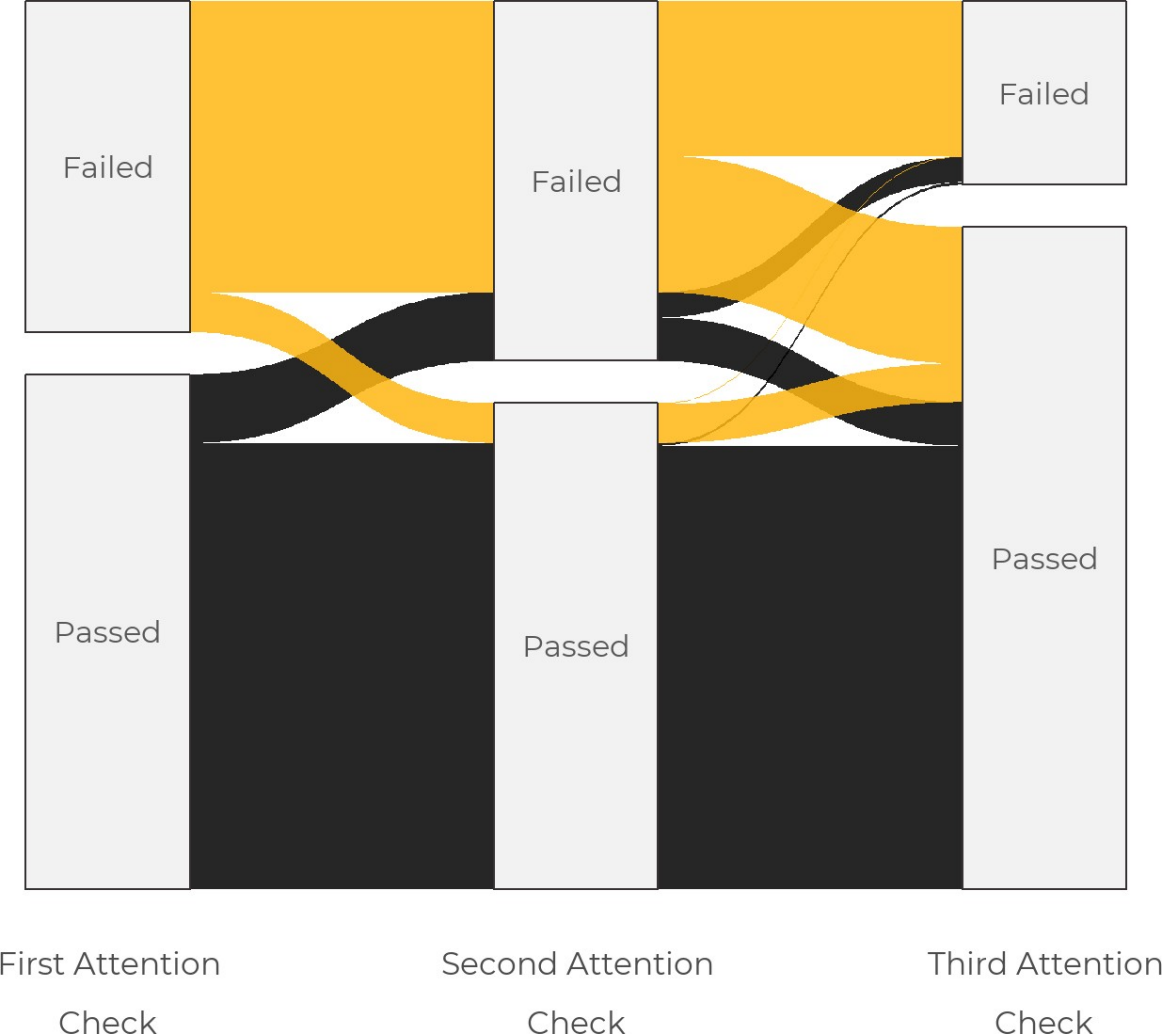
Distribution of multiple attempts in the survey across countries.

Fig 2 presents the magnitude of multiple responses from the young adults who participated in the survey based on the IP address logged on the survey website. As presented in Fig 2, less than 10% of the responses received were from subsequent multiple attempts of the survey, with a higher percentage of multiple attempts from responses received from Nigeria. About 9% of survey responses received from participants in Nigeria were second or higher-ordered compared to about 3% in Kenya and 2% in South Africa.

### Demographics of participants

A summary description of participants recruited in the survey is presented in Table 4. The mean age of the participants ranged from about 21.6 years in Kenya to 20.0 years in Nigeria. There were no noticeable differences in the gender of participants in Kenya (SR: 82 young men participants per 100 young women) and Nigeria (SR: 97 young men/100 young women). However, in South Africa, about 68% of the participants recruited were women, while only about 32% were men. Participants were mostly Black/Africans. More than half of the participants had attained tertiary or higher education in all countries, with a fair representation of participants with less than tertiary education in South Africa (47%) and Nigeria (38%). About 40% of the participants in Kenya and 46% of young adults from South Africa were not married but in a relationship, while 62% of participants in Nigeria were not married nor in a relationship. More than 70% of the participants in all countries reported using the internet every day.

**Table 4:** Sociodemographic characteristics of survey participants.

Social Demographic Characteristics	Kenya N = 410	Nigeria N = 278	South Africa N = 248	Total N = 936
Age	21.6 (1.77)	20.0 (1.72)	20.5 (1.82)	20.8 (1.90)
Gender:				
Female	225 (54.9%)	141 (50.7%)	168 (67.7%)	534 (57.1%)
Male	185 (45.1%)	137 (49.3%)	80 (32.3%)	402 (42.9%)
Race/Ethnicity:				
Black/African	407 (99.3%)	271 (97.5%)	234 (94.4%)	912 (97.4%)
Others	3 (0.73%)	7 (2.52%)	14 (5.65%)	24 (2.56%)
Highest Educational Attainment:				
< Tertiary	121 (29.5%)	105 (37.8%)	116 (46.8%)	342 (36.5%)
Tertiary/Higher	289 (70.5%)	173 (62.2%)	132 (53.2%)	594 (63.5%)
Relationship Status:				
Not married: not in relationship	162 (39.5%)	171 (61.5%)	115 (46.4%)	448 (47.9%)
Not married: in a relationship	205 (50.0%)	106 (38.1%)	124 (50.0%)	435 (46.5%)
Married/Living with Partner	43 (10.5%)	1 (0.36%)	9 (3.63%)	53 (5.66%)
Frequency of Internet Use:				
Everyday	300 (73.2%)	204 (73.4%)	195 (78.6%)	699 (74.7%)
Sometimes	89 (21.7%)	61 (21.9%)	36 (14.5%)	186 (19.9%)
Occasionally/Rarely	21 (5.12%)	13 (4.68%)	17 (6.85%)	51 (5.45%)
Quality Assessments				
Time Spent to Complete Survey (Mins)	14.2 (5.39)	12.6 (5.32)	13.6 (5.10)	13.6 (5.34)
Total Number of Attention Checks Passed:				
0	78 (19.0%)	40 (14.4%)	53 (21.4%)	171 (18.3%)
1	75 (18.3%)	55 (19.8%)	50 (20.2%)	180 (19.2%)
2	42 (10.2%)	25 (8.99%)	27 (10.9%)	94 (10.0%)
3	215 (52.4%)	158 (56.8%)	118 (47.6%)	491 (52.5%)

### Attentiveness to survey

**Fig 3:**
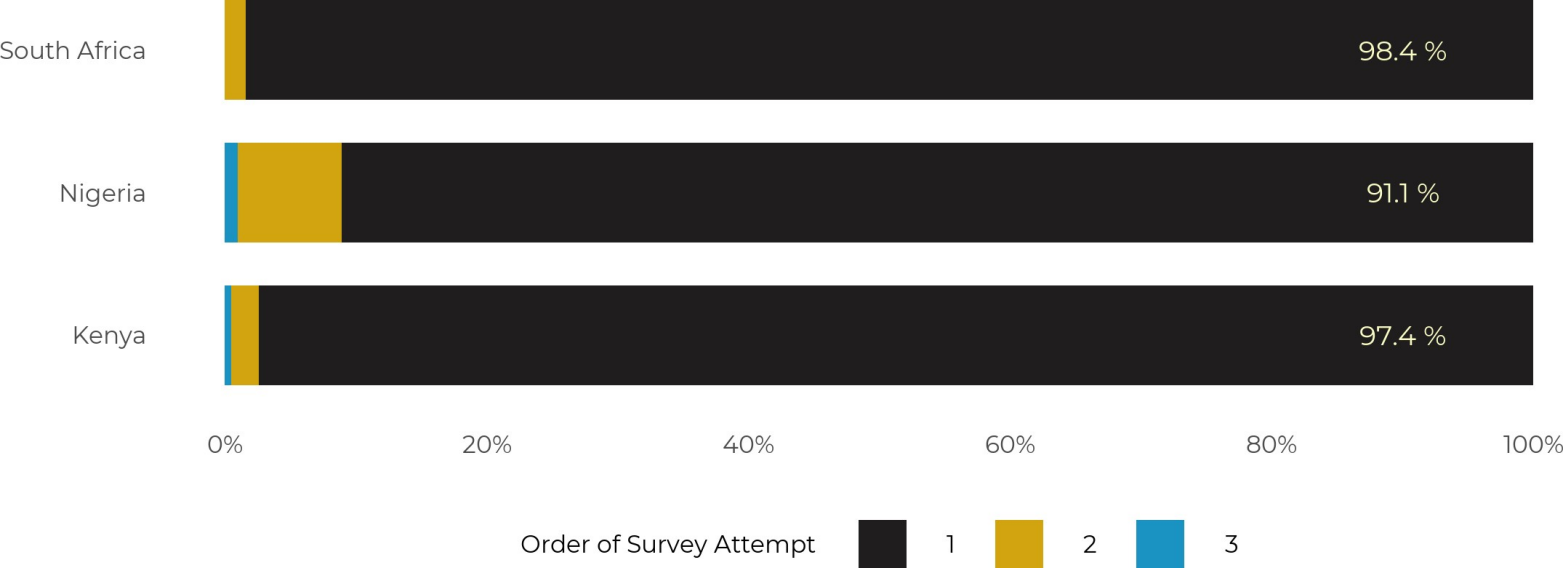
Passage rates across the three attention check questions.

The average time to complete the survey varied across the countries from about 14 minutes in Kenya ($\bar{x}$ = 14.2 mins) and South Africa ($\bar{x}$ = 13.6 mins) to about 13 minutes ($\bar{x}$ = 12.6 mins) in Nigeria. About half of the participants from Kenya (52%) and Nigeria (57%) passed all attention checks, while only about 48% of the young adults from South Africa passed all attention check questions. In addition to the distribution of the total number of attention checks passed by the participants across countries, efforts were also made to assess how participants’ attention wanes across the survey items. Fig 3 shows the aggregate passage rate and the patterns of passing the three attention checks. The figure shows that the passage rate on attention checks varied greatly from about 50% on the first attention check to as high as 76% on the third attention check question. The majority of those who passed the first attention check also passed the second and most of those who passed the second attention check passed the last question. Surprisingly, a significant percentage of the participants who failed the first two attention checks passed the last attention check question.

### Patterns of passage of attention checks by sociodemographics

Passage rates for each level of attention check passed across sociodemographic characteristics are presented in Table 5. About 57% of young women passed all attention checks compared to 47% of young men in the sample. More participants from other racial groups (58%) also passed three attention-check questions compared to young Black/African adults (52%). About 57% of the participants who were married or living with a partner passed all the attention check questions compared to about 54% of those who were not married nor in a relationship and 50% of those who were not married but in a relationship. About half of those who use the internet every day (52%) or sometimes (58%) passed all attention check questions compared to about 39% of those who reported using the internet occasionally or rarely. Notable differences were also observed in time spent in completing the survey. Those who failed to pass all the attention checks spent an average of 12 minutes ($\bar{x}$ = 11.8 mins) compared to about 14 minutes ($\bar{x}$ = 14.3 mins) among those who passed two or all the attention check questions.

**Table 5:** Passage rates of attention check questions across sociodemographic characteristics.

Sociodemographic Characteristics	Number of Attention Checks Passed			
	0 N = 171	1 N = 180	2 N = 94	3 N = 491
Age	21.0 (1.86)	21.1 (1.80)	20.7 (1.94)	20.7 (1.92)
Gender:				
Female	86 (16.1%)	95 (17.8%)	51 (9.55%)	302 (56.6%)
Male	85 (21.1%)	85 (21.1%)	43 (10.7%)	189 (47.0%)
Race/Ethnicity:				
Black/African	168 (18.4%)	174 (19.1%)	93 (10.2%)	477 (52.3%)
Others	3 (12.5%)	6 (25.0%)	1 (4.17%)	14 (58.3%)
Highest Educational Attainment:				
< Tertiary	66 (19.3%)	66 (19.3%)	35 (10.2%)	175 (51.2%)
Tertiary/Higher	105 (17.7%)	114 (19.2%)	59 (9.93%)	316 (53.2%)
Relationship Status:				
Not married: not in relationship	74 (16.5%)	89 (19.9%)	43 (9.60%)	242 (54.0%)
Not married: in a relationship	91 (20.9%)	83 (19.1%)	42 (9.66%)	219 (50.3%)
Married/Living with Partner	6 (11.3%)	8 (15.1%)	9 (17.0%)	30 (56.6%)
Frequency of Internet Use:				
Everyday	128 (18.3%)	139 (19.9%)	69 (9.87%)	363 (51.9%)
Sometimes	29 (15.6%)	30 (16.1%)	19 (10.2%)	108 (58.1%)
Occasionally/Rarely	14 (27.5%)	11 (21.6%)	6 (11.8%)	20 (39.2%)
Country of Residence:				
Kenya	78 (19.0%)	75 (18.3%)	42 (10.2%)	215 (52.4%)
Nigeria	40 (14.4%)	55 (19.8%)	25 (8.99%)	158 (56.8%)
South Africa	53 (21.4%)	50 (20.2%)	27 (10.9%)	118 (47.6%)
Time Spent to Complete Survey (Mins)	11.8 (4.81)	13.0 (5.41)	14.3 (5.76)	14.3 (5.23)

Multiple regression models were fitted on the data to further delineate statistically significant differences in the passage rates for the attention checks across sociodemographic characteristics. The results of the models are presented in Table 6. The first column (a) presents results from a truncated Poisson regression model based on the number of attention checks passed by each participant. The results presented in Table 6(A) suggest that the passage rate was significantly different by age, sex, marital status, frequency of internet use, and completion time. Higher age [IRR = 0.94, 95%CI: 0.92–0.97] was associated with passing fewer attention checks. Young men [IRR = 0.82, 95%CI: 0.76–0.89] were also significantly less likely to pass a higher number of attention checks compared to the young women who participated in the survey. Married participants or those living with a partner [IRR = 1.26, 95%CI: 1.05–1.51] passed more attention checks compared to those who were not married or in a relationship. Participants who used the internet occasionally [IRR = 0.78, 95%CI: 0.64–0.95] were also significantly less likely to pass more attention checks compared to those who use the internet every day. Across the countries, participants from Nigeria [IRR = 1.08, 95%CI: 0.97–1.20] did not significantly differ from those from Kenya in terms of the number of attention checks passed while participants from South Africa [IRR = 0.87, 95%CI: 0.78–0.97] passed less attention check questions compared to those from Kenya. Furthermore, a statistically significant association was observed between the time spent completing the survey and the number of attention checks passed. An increase in the number of minutes spent on the survey [IRR = 1.03, 95%CI: 1.02–1.04] was significantly associated with passing more attention check questions while adjusting for covariates.

**Table 6:** Multivariate regression models showing associations between the passage of checks passed and sociodemographic characteristics.

Characteristics	(a) Number of Screeners Passed		Complementary Log Log Regression Model					
			(b) Passed = 0 vs. Passed ≥ 1		(c) Passed ≤ 1 vs. Passed ≥ 2		(d) Passed ≤ 2 vs. Passed = 3	
	IRR	CI	exp (β)	CI	exp (β)	CI	exp (β)	CI
Age	0.94***	0.92 0.97	0.96	0.91 1.01	0.90***	0.85 0.95	0.91**	0.86 0.97
**Gender**	** **	** **						
Female	Reference		Reference		Reference		Reference	
Male	0.82***	0.76 0.89	0.85	0.72 1.01	0.78**	0.65 0.93	0.73**	0.6 0.88
**Race**	** **	** **						
Black/African	Reference		Reference		Reference		Reference	
Others	1.22	0.94 1.58	1.40	0.8 2.41	1.19	0.66 2.01	1.39	0.76 2.35
**Educational Attainment**								
Secondary	Reference		Reference		Reference		Reference	
Tertiary/Higher	1.08	0.99 1.18	1.09	0.91 1.3	1.13	0.94 1.37	1.11	0.91 1.36
**Marital Status**		** **						
Not Married: Not in a relationship	Reference		Reference		Reference		Reference	
Not married: in a relationship	0.95	0.87 1.03	0.88	0.74 1.05	0.93	0.78 1.12	0.93	0.77 1.13
Married/Living with Partner	1.26*	1.05 1.51	1.41	0.95 2.08	1.59*	1.08 2.31	1.27	0.83 1.88
**Frequency of Internet Use**								
Everyday	Reference		Reference		Reference		Reference	
Sometimes	1.05	0.95 1.17	0.99	0.8 1.23	1.06	0.85 1.32	1.09	0.86 1.36
Occasionally/Rarely	0.78*	0.64 0.95	0.76	0.51 1.09	0.71	0.46 1.05	0.68	0.41 1.04
**Country of Residence**								
Kenya	Reference		Reference		Reference		Reference	
Nigeria	1.08	0.97 1.2	1.22	0.98 1.51	1.06	0.85 1.32	1.09	0.86 1.38
South Africa	0.87*	0.78 0.97	0.89	0.72 1.11	0.82	0.65 1.03	0.79	0.62 1.01
**Completion Time (Mins)**								
Completion Time (Mins)	1.03***	1.02 1.04	1.05***	1.03 1.06	1.04***	1.03 1.06	1.04***	1.02 1.05
**Intercept**	6.06***	3.59 10.23	2.43	0.83 7.1	5.31**	1.72 16.41	3.36*	1.02 11.16

**Note:** ***p < .*001;*

**p < .*01;*

*p < .*05;*

**IRR** = Incidence rate ratios *[values greater than 1 implies a higher likelihood*, *values less than 1 implies a lower likelihood];* **exp(β)** = Exponentiated Coefficient *[values greater than 1 implies a higher likelihood*, *values less than 1 implies a lower likelihood];* **CI** = 95% Confidence Intervals.

Table 6(B)–6(D) also presents results from a complementary-log logistic regression model to evaluate how socioeconomic characteristics vary across varying thresholds of the number of attention checks passed. The results from model (b) showed that the likelihood of passing at least one attention check did not significantly differ across the key sociodemographic characteristics such as age, sex, education, or marital status. On the contrary, key demographic characteristics like age and sex were significantly associated with passing at least two attention check questions (model c) and passing all three attention check questions (model d). Married participants or those living with a partner [exp(β) = 1.59, 95%CI: 1.08–2.31] were also more likely to pass two or more attention-check questions compared to young adults who were not married nor in a relationship. Interestingly, the amount of time spent (in minutes) on the survey was associated with the number of attention checks passed at varying thresholds and in the same direction. A higher amount of time spent on the survey was associated with passing at least one attention check [exp(β) = 1.05, 95%CI: 1.03–1.06], passing at least two attention checks [exp(β) = 1.04, 95%CI: 1.03–1.06] and passing all attention checks [exp(β) = 1.04, 95%CI: 1.02–1.05].

## Discussion

In this study, I presented one of the first comprehensive analyses of the efficiency and effectiveness of recruiting young adults in Kenya, Nigeria, and South Africa via the Facebook advertising platform. With this study, I have made three important contributions to scholarship. First, I showed that Facebook advertising offers a promising opportunity to effectively recruit young adults (even those with limited internet access) in Kenya, Nigeria, and South Africa at a reasonable cost and within a short period. The cost of advertising for this group was relatively lower than in some of the previous studies. For example, a study of young women from Victoria, Australia, reported spending about $0.67 per link click, amounting to $10.16 per expression of interest, or $20.14 per complaint participant [3]. While the recruitment costs are hardly comparable based on several factors, the evidence suggests that Facebook recruitment is cost-effective in reaching young adults [2–5]. The match rates were also relatively high in this study, although moderately lower than those reported in another study [1,65,76]. The high match rates could have been because the advertisements targeted participants based on key demographic characteristics such as age, sex, and country, all of which have been associated with higher match rates in previous studies [65,76].

Secondly, the analysis revealed that the Facebook sample is not inherently free from inattentive or poor-quality responses. I found that about half of the participants passed all attention checks. This passage rate is comparable to those observed by Berinsky et al. [51] and Oppenheimer et al. [48]. Among pregnant women recruited via Mturk, about 75% answered all three questions correctly, while about 2% missed or skipped all three attention checks [77]. Careless responding also ranged from 8% in study 1 by Hauser and Schwarz [58], 24% in study 1 by Gummer et al. [53], and up to as much as 78% in Mancosu et al. [71]. Between a third and a half of the respondents in a national sample failed to accurately answer an attention check question [51].

Prior studies have shown that when the incentive is low for survey takers to provide careful responses (such as pay per complete, consistent response), careless responding tends to be higher [61]. Explicit warnings such as “responding without much effort will be flagged for low-quality data” [56] and “responding without much effort would result in loss of credits” were shown to increase quality responses [57]. Although an incentive was offered to winners selected randomly in this study, there was no mention that the selection of the winners would be based on passing the attention checks. This may have contributed to the higher level of nonattention in this study. More so, in an incentivized study, participants may want to rush through the survey to collect the incentive as has been suggested [51]. I also found some positive effects of attention checks. It appears that survey quality could improve as a survey progresses. It emerged that fewer participants failed the last attention check than the first two. More so, most of the participants who passed the second attention check question passed the last attention check. Similar to passage rates reported in this study, Clifford and Jerit [45], using two attention check questions, found that 38% of their respondents passed the first attention check while 62% passed their last item.

Some prior studies have highlighted that excluding participants based on the passage of attention checks could introduce some demographic bias and advise excluding those who fail an attention check only when those who fail and those who pass are relatively similar [48]. For example, Berinsky et al. [51] suggested that an attention check question could be associated with sociodemographic characteristics such as education and race. I evaluated this pattern of bias using the Facebook sample. It appeared that those who failed and those who passed were relatively similar in terms of race and education but not age and sex. I found significant associations between demographic characteristics—age and sex—and passing a higher number of attention check questions. Men and those who were older were significantly less likely to pass more attention check questions. This finding is contrasts to another study that those who failed attention check questions tended to be younger and less educated than those who passed [62]. It is, however, worthy of note that older adults in this study include those who were older than 24 years and under 45 years, thus limiting the comparability of these studies. However, it is likely that the slightly older young adults in the sample were more distracted while participating in the survey or raced through the survey.

The results, further stratified at different thresholds of the number of attention checks passed, suggest that excluding inattentive participants who failed to pass at least two attention check questions could lead to the under-representation of older young adults and young men. I also found a positive association between inattentive responding and time spent to complete the survey. Higher passage rates on the attention check questions were significantly associated with higher cumulative time spent taking the survey. This finding is consistent with prior work that used the amount of time spent on a survey as a measure of respondents’ efforts [51,57,78]. These studies have shown a positive association between the number of attention checks passed and the time spent to complete a survey. Greszki et al. [55] suggested that respondents who superficially perform the cognitive task of answering questions could be expected to complete a survey very quickly because speeding could be considered to indicate that respondents devoted little attention to processing and answering questions. Gummer et al. [53] also found that those who failed the IRI were more likely to speed through the survey than more attentive participants. In another study, participants who failed the IMC took less time to complete the experiment and were reliably lower in need for cognition than those who passed [48].

However, filtering inattentive based on response time alone may not be a good metric for assessing the quality of responses. This may especially be the case in African countries where internet penetration and strength of connection differ across countries, cities, and towns. Moreover, a recent study concluded that filtering inattentive respondents based on response times could introduce some bias unless the study is largely a replication of known results with a validated expected completion time [50]. As highlighted in this study, response time to complete the survey varied significantly across the studied countries, with a higher response time among young adults from Kenya and a lower response time from young adults from Nigeria. This may especially be because response time to complete a survey among young African adults could be influenced by the type of device, browser, and the strength of internet connection. As a result, nonattentive participants could equally take longer to complete a survey if webpages take longer to load.

While this study contributes to the literature in diverse ways, it is not without limitations. The first is that the restriction of incentives to only a few “lucky” winners could have limited the recruitment size since some participants may weigh up the effort and probability of winning. Perhaps future studies could explore the effects of different phrasings for offers of incentives on recruitment rates, competition rates, and attentiveness. Secondly, it is not completely clear the extent to which the inactivity of the group may have affected the recruitment rate since page activity is likely to increase the reputation of the survey. Nevertheless, I was available to answer participants’ questions and provided a mobile number for participants to contact me should there be a need. Lastly, the generalizability of the study findings is limited to young adults who have access to the internet and have a presence of social media. Despite these limitations, this work paves the way for future studies to advance the use of online samples in population studies, especially in developing countries. Although not explored in detail in this study, the use of Facebook Pixel for tracking conversions to the survey offers yet another fantastic opportunity for researchers to retarget study participants. According to Facebook, advertisers could retarget members of a demographic who performed specific actions on the survey website. Leveraging this tool could open a window of opportunity for researchers to lead large-scale representative online panel surveys using the advertising platform or even experimental research by setting controls based on lookalike audiences on the advertisement manager [79].

## Conclusion

Throughout this article, I have presented evidence for the sustained use of the Facebook advertising platform in recruiting young adults in African countries and highlighted important considerations for the quality of responses. This is especially a timely piece given the recent advances and increasing interest in online surveys. In the first part, I provided evidence that Facebook advertising is cost-effective and efficient in reaching and recruiting young adults based on targeted characteristics in a short period of time. In the second part, I uncovered important considerations and showed that Facebook samples are not inherently free from careless responding and that as many as half of the respondents behaved in this manner. More importantly, I demonstrated that attention checks help to distinguish nonattentive from attentive participants. Also, I found evidence that the passage of attention checks in the sample correlates with demographically relevant characteristics such as age and sex.

Researchers must be cognizant of these differences and careful when excluding inattentive respondents as this may skew the sample and induce bias. While excluding participants based on attention checks could introduce some bias, as I have shown in this study and subsequently lead to the over-representation of some groups, I believe that the importance of attention checks far outweighs its limitations. Attention checks allow researchers to conclude with relatively high confidence that respondents have carefully or not carefully processed and responded to a survey question. Although this study justifies the use of attention checks to ensure data quality, it also raises significant concerns for how researchers might deal with inattentive respondents while ensuring the validity of responses. There is clearly more work to be done, and future studies could examine if the use of non-response weights based on the excluded participants or post-stratification weights increases the external validity of studies excluding participants who devote less attention to the survey.

## Supporting information

S1 Appendix Survey questionnaire. (PDF)
