# Supplementary material for: Characterizing low effort responding among young African adults recruited via Facebook advertising
Source: PLoS One. 2021 May 14;16(5):e0250303. doi: 10.1371/journal.pone.0250303 (PMC8121367; doi:10.1371/journal.pone.0250303)
Supplement: S1 Appendix — (PDF) [file pone.0250303.s001.pdf]

Appendix A: Survey Wording

This appendix provides additional detail on the exact wording of the attention check (AC) questions and other questions – social characteristics (SC) and demographic characteristics (DC). The survey was part of a large-scale study for understanding the health information needs of young adults in African countries. This Appendix presents only a section of the entire questionnaire.

No.	Questions and instructions	Coding Categories
DC.01	How old were you at your last birthday?	
DC.02	Gender:	☐ Male<br>☐ Female
DC.03	In what country are you currently residing?	
SC.01	What is your race or ethnicity?	☐ Black/African<br>☐ White<br>☐ Coloured/Mixed Race<br>☐ Indian/Asian<br>☐ Others
SC.02	How often do you use the internet?	☐ Rarely - Less than once per week<br>☐ Occasionally - At least once a week<br>☐ Sometimes - in about 50% of the chances when I could have<br>☐ Every day and Every time<br>☐ Not Sure
SC.03	What is the highest level of education that you have attained	☐ Never attended school<br>☐ Primary<br>☐ Secondary<br>☐ Higher/Tertiary<br>☐ Prefer Not to Say
SC.04	What is your current relationship status	☐ Not married: not in a relationship<br>☐ Not married: in a relationship<br>☐ Not married: living with a partner as if married<br>☐ Currently married<br>☐ Formerly married<br>☐ Prefer Not to Say

AC.01	*To what extent do you agree or disagree with the statement below? Note that this question is an attention checker to gauge that you are not distracted.*<br><br>You should only select the option that has **"green"** to confirm that you are attentive to the questions.	☐ Very Strongly Agree<br>☐ Strongly Agree<br>☐ Agree<br>☐ Neutral<br>☐ Disagree<br>☐ *Strongly Disa green*<br>☐ Very Strongly Disagree
AC.02	*To what extent do you agree or disagree with the statement below? Note that this question is an attention checker to gauge that you are not distracted.*<br><br>This question is an attention checker. You should select the option that has **"red"** to confirm that you are attentive to the questions.	☐ Very Strongly Agree<br>☐ *Strongly Ag red*<br>☐ Agree<br>☐ Neutral<br>☐ Disagree<br>☐ Strongly Disagree<br>☐ Very Strongly Disagree
AC.03	*To what extent do you agree or disagree with the statement below? Note that this question is an attention checker to gauge that you are not distracted.*<br><br>This question is an attention checker. You should only select the option that has **"grey"** as your response.	☐ Very Strongly Agree<br>☐ Strongly Agree<br>☐ *A grey*<br>☐ Neutral<br>☐ Disagree<br>☐ Strongly Disagree<br>☐ Very Strongly Disagree
